# Emerging Trends in the Treatment of Skin Disorders by Herbal Drugs: Traditional and Nanotechnological Approach

**DOI:** 10.3390/pharmaceutics16070869

**Published:** 2024-06-28

**Authors:** Rutvi Agrawal, Priyanka Jurel, Rohitas Deshmukh, Ranjit Kumar Harwansh, Akash Garg, Ashwini Kumar, Sudarshan Singh, Ajay Guru, Arun Kumar, Vinoth Kumarasamy

**Affiliations:** 1 Rajiv Academy for Pharmacy, Mathura 281001, Uttar Pradesh, India; agrawalrutvi96@gmail.com (R.A.); akashgarg983@gmail.com (A.G.); 2 Institute of Pharmaceutical Research, GLA University, Mathura 281406, Uttar Pradesh, India; priyankasinghjurel@gmail.com (P.J.); rahi18rahi@gmail.com (R.D.); harwanshranjeet@gmail.com (R.K.H.); 3 Research and Development Cell, Department of Mechanical Engineering, School of Engineering and Technology, Manav Rachna International Institute of Research and Studies, Faridabad 121003, Haryana, India; drashwinikumar.research@gmail.com; 4 Faculty of Pharmacy, Chiang Mai University, Chiang Mai 50200, Thailand; sudarshansingh83@hotmail.com; 5 Office of Research Administration, Chiang Mai University, Chiang Mai 50200, Thailand; 6 Department of Cariology, Saveetha Dental College and Hospital, Saveetha Institute of Medical and Technical Sciences, Saveetha University, Chennai 600077, Tamil Nadu, India; ajayguru.sdc@saveetha.com; 7 School of Pharmacy, Sharda University, Greater Noida 201306, Uttar Pradesh, India; 8 Department of Parasitology and Medical Entomology, Faculty of Medicine, Universiti Kebangsaan Malaysia, Jalan Yaacob Latif, Cheras, Kuala Lumpur 56000, Malaysia

**Keywords:** skin, herbal formulations, skin diseases, drug delivery system, nanoparticles, ethosomes

## Abstract

Since the earliest days, people have been employing herbal treatments extensively around the world. The development of phytochemical and phytopharmacological sciences has made it possible to understand the chemical composition and biological properties of a number of medicinal plant products. Due to certain challenges like large molecular weight and low bioavailability, some components of herbal extracts are not utilized for therapeutic purposes. It has been suggested that herbal medicine and nanotechnology can be combined to enhance the benefits of plant extracts by lowering dosage requirements and adverse effects and increasing therapeutic activity. Using nanotechnology, the active ingredient can be delivered in an adequate concentration and transported to the targeted site of action. Conventional therapy does not fulfill these requirements. This review focuses on different skin diseases and nanotechnology-based herbal medicines that have been utilized to treat them.

## 1. Introduction

The skin is the largest organ of the human body. The skin acts as the initial defense line. The skin has several different cells and structures. The three main layers are the dermis, hypodermis, and epidermis. Every layer has a different role to play in the overall functionality of the skin. Every layer on the body varies in thickness. It is the thickest on the palms and soles and thinnest on the eyelids. The position of the skin affects the dermis’s thickness as well. Beneath the dermis is the hypodermis or subcutaneous connective tissue. The subcutaneous layer varies in thickness from person to person and throughout the body. The primary skin appendages are sweat glands, oil glands, and hair follicles. The skin covers and protects the interior organs, bones, muscles, and ligaments. Hairy skin and glabrous skin are the two basic types of skin [1]. The skin can also be pale, sensitive, dry, sagging, and worn out.

Skin is essential for defending the body from infections as well as water loss [1,2]. Other roles include water resistance, insulation, temperature control, sensations, storage, and vitamin D synthesis by ultraviolet (UV) light. It also protects vitamin B folates and aids in the absorption of various medications [3]. Scar tissue formation is an attempt by skin to heal from severe wounds. Frequently, this is depigmented and dingy [4].

For both the treatment and prevention of numerous diseases, natural products and conventional pharmaceuticals are beneficial [5]. They are becoming more popular as complementary therapies for typical skin diseases [6]. Plant-based natural medicines have grown in popularity throughout time because of their many advantages, such as few side effects, low cost, and long-term acceptability. Additionally, medicinal plants can be used as raw materials in the creation of novel therapeutic medicines. *Calendula officinalis*, *Azadirachta* A. Juss., *Portulaca amilis*, *Aloe vera*, and many more plants have been studied for the treatment of various skin conditions ranging from itching to skin cancer and they have been reported to be effective in managing a number of skin conditions [4,7]. Numerous traditional medical systems, including Chinese Medicine, Ayurveda, Siddha, and the Unani System of Medicine, have extensive documentation on the therapeutic use of plants. Nearly a third part of the most popular medications in the market today come from plants or alternative natural sources [8,9]. Herbal medications have unique properties that conventional drug administration methods like capsules, syrups, and decoctions cannot match. Nonetheless, using nanotechnology to deliver herbal medications has proven to be highly effective [10].

Herbal technology has proven useful in a number of medical sciences sectors, including organ and tissue imaging, artificial implants, nanorobotics, biosensors, and enhanced drug delivery systems, such as regulated or sustained drug delivery. Herbal nanomedicines are nanosized pharmaceuticals that include extracts, concentrated fractions, or biomarker elements of herbal medications. Due to their higher bioavailability and lower toxicity, herbal nanomedicines possess a number of advantages [11]. With improved bioavailability and fewer toxicities, combining herbal medicine with nanotechnology may be a valuable resource for the development of herbal formulations [12]. Overall, it is well-established that herbal nanomedicines are safer, more bioavailable, and have greater therapeutic value than traditional herbal and synthetic pharmaceuticals [11].

Although the contributions of nanotechnology are helpful in a number of medical fields, it is important to draw attention to specific drawbacks. Common concerns related to nano-sized particles including the potential to cause hazardous lung diseases and lead to other diseases that might cause changes in homeostasis or even mortality, have been cited by clinical researchers [13]. Other negative factors mentioned include difficulties in scaling up procedures and high cost [14]. This review outlines the application of several herbal nanoformulations, along with different skin conditions and herbal drugs used to treat them. Additionally, it shows several patents that have been published on skin conditions. This paper explains how the popularity of herbal products has increased interest among researchers and enhanced productivity by preventing several skin conditions.

## 2. Skin Diseases

**Figure 1:**
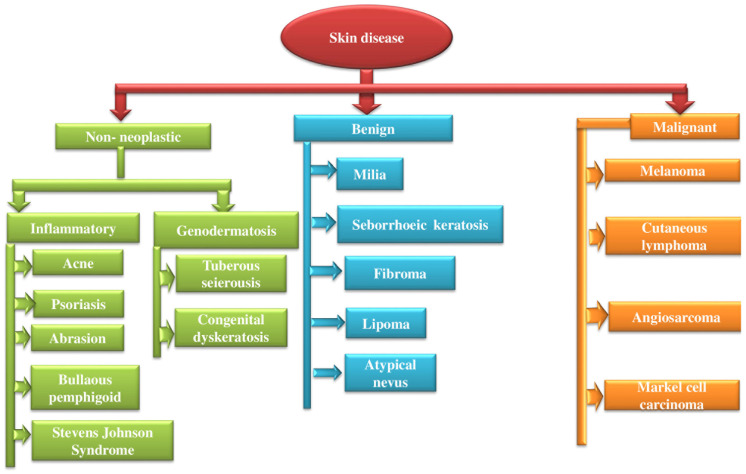
Various types of skin diseases. Reproduced with permission from [18] Creative Commons Attribution License 4.0.

The human body’s largest and most varied organ is the skin. The general state of one’s skin is significant for both cosmetic and health reasons. The patient’s psychological state is impacted by its unfavorable appearance due to dermatitis, and both of these elements are crucial in the development and management of chronic skin illnesses [15]. Increased awareness of one’s body and appearance, especially among young people, exacerbates anxiety [16]. A number of variables, including gender, race, personal cleanliness, the standard of skincare, the environment, and diet, influence the occurrence and incidence of cutaneous illnesses in young people [17]. In certain cases, patients seem to manifest lesions of the skin due to the tensions brought on by interpersonal disputes and unresolved emotional issues [16]. The different skin diseases are shown in Figure 1 [18].

### 2.1. Common Skin Problems

Skin conditions are a prevalent ailment that can injure a person in various ways and affect people of all ages. Despite the fact there are over more than one thousand illnesses that can damage the skin, the main categories of skin diseases are discussed below [4]. 

#### 2.1.1. Rashes

A rash is a collection of several patches or a specific area of irritated skin surface. It can be brought on by infections, allergies, and overactive oil glands, which can result in psoriasis, dermatitis, eczema, and acne [19].

#### 2.1.2. Viral Infections

Viral skin disorders affect persons of all ages and range from straightforward exanthems to intricate systemic illnesses. Most exanthems of viral origin are self-limiting. Warts, herpes simplex, and herpes zoster are a few examples of viral skin infections [20].

#### 2.1.3. Bacterial Infections

Acute bacterial and skin structure infections are two categories of bacterial skin infections, along with skin and soft tissue infections. Both cellulitis and erythrasma are complicated bacterial illnesses that are impacted by fat. Other complex bacterial illnesses include erysipelas, wound infections, and severe cutaneous abscesses.

#### 2.1.4. Fungal Infection

Moisture and maceration in intertriginous zones create a perfect habitat for yeasts to grow, including Candida species. This causes inflammation and redness, as well as satellite lesions in the inguinal area, beneath the breasts, and in the abdominal folds. An important part of the growth of candidiasis is friction and maceration. Although less common, obese people can also have candidal folliculitis. While oral medications like fluconazole or itraconazole or topical antifungals like ketoconazole and econazole creams can effectively treat the illness, they cannot stop recurrences.

#### 2.1.5. Pigmentation Problems

Patients with abnormal pigmentation, such as those with vitiligo, melasma, and post-inflammatory hyperpigmentation (PIHP), may also have serious concerns. Pigment loss, commonly in the lips and fingertips, is called vitiligo. Melasma, which is characterized by symmetrical regions of hyperpigmentation, can result from a variety of factors, such as exposure to high UV radiation levels, chemical agents, female hormone imbalances, or genetic abnormalities [21]. PIHP is commonly associated with skin inflammation and may manifest during surgery, laser ablation, dermatitis flare-ups, or in patients treated with photosensitizing medications [22,23].

#### 2.1.6. Cancers

Skin cells can be harmed by excessive sun exposure or other UV radiation, leading to the development of skin cancer. Skin cancer can be classified into three primary types: melanoma, squamous cell carcinoma (SCC), and basal cell carcinoma (BCC). Even though melanoma is the rarest form of skin cancer, its high rate of metastasis makes it the deadliest. However, keratinocyte malignancies, such as BCC and SCC, account for the majority of cases of skin cancer [24].

#### 2.1.7. Others

Among the problems that cannot be clearly characterized are wrinkles, rosacea, spider veins, and varicose veins. The breakdown of collagen and elastin in the dermis results in drooping skin, ultimately giving rise to wrinkles. Rosacea is a chronic condition that causes the skin of the human face to grow red, develop lesions and pimples, and, less frequently, expand the nose. Its origin is a mystery. When blood vessels develop and become apparent through the skin’s surface, spider veins and varicose veins are evident [4]. Erythema is the outcome of an inflamed cutaneous reaction linked to conditions including psoriasis, acne, fever from systemic disorders, and sunburn from exposure to certain electromagnetic wave bands, primarily ultraviolet radiation [22].

## 3. Herbal Drugs for Skin Diseases

The use of synthetic molecules and chemical compounds can result in skin toxicity from safety concerns with long-term use. The demand for herbal drugs has skyrocketed due to an increased awareness of the photoaging and carcinogenic consequences and socioeconomic advancement [25,26]. Overall, there is an increase in demand for plant-based medications, health goods, food supplements, and cosmetics. The cause of this is the rising understanding that natural products are harmless, have few adverse effects, and are widely accessible at low costs [27]. All scientific communities are concentrating their study on understanding the chemical constitution of plants with medicinal properties and their common usage. With fewer adverse effects than current medications, this research could result in increasingly ingenious products [14,28].

According to the World Health Organization, herbal medications are fully developed and labeled pharmaceuticals that contain combinations of aerial, subterranean, or other plant elements, as well as active ingredients [27]. Natural drugs made from plants are becoming increasingly popular because of a number of advantages, such as frequently fewer side effects, increased patient compliance, being comparatively cheaper, and being widely accepted due to a long history of use. Herbal treatments also provide sensible approaches to address a range of ailments that are incurable or difficult to cure with traditional medical procedures. These factors have led to studies on a variety of plants as possible remedies for skin disorders ranging from skin cancer to itching. Over the previous years of research, more than 31 plants have so far been reported to be beneficial in treating a variety of skin conditions [4]. Various herbal drugs used for different skin diseases are discussed below.

### 3.1. Atopic Dermatitis

An inflammatory, itchy, and chronic skin condition is known as atopic dermatitis [29].

- Licorice

A thorough analysis summarizes the anti-inflammatory properties of licorice, i.e., *Glycyrrhiza glabra* L. and *Glycyrrhiza uralensis*. Glycyrrhetinic acid, the triterpenes of licorice, and glycyrrhizin were used in the skin investigations [30,31]. Nevertheless, several components, such as the chalcone licochalcone A [32] and the flavonoid isoliquiritigenin [33], also have anti-inflammatory properties. Licochalcone A-containing cream showed better anti-inflammatory effects in placebo-controlled research including 26 individuals [34]. Furthermore, a placebo-controlled double-blind UV-erythema evaluation study demonstrated anti-inflammatory effects with an herbal composition, containing 0.1% root extract of *Glycyrrhiza uralensis* and 0.6% glycyrrhizinic acid as the main active ingredients, at 48 h after irradiation and cream application. The medication made of licorice was just as successful as 1% hydrocortisone acetate. A non-interventional pilot study also resulted in a reduction in the severity score in ten atopic dermatitis patients given this medication twice daily for two weeks [35].

b. Tormentil and Evening Primrose

Since ancient times, dermatologists have empirically employed tannins from black tea and oak bark. Tannins are applied topically as local baths or wet-lipid wraps to treat oozing and acute eczema [36]. Due to the high γ-linolenic acid content in evening primrose oil, it is good for atopic dermatitis. It finds applications in topical products as well as in products for internal use [36]. After 48 h, an occlusive patch test revealed that a cream with 2% tannins obtained from the roots of tormentil had a vasoconstrictive effect similar to that of a corticoid [37].

### 3.2. Psoriasis

Herbal medicines are also applied topically to treat psoriasis. Long-term immune-mediated psoriasis is characterized by scaly, red, burning, and itchy skin patches [36].

- Araroba tree

The anthracene derivative dithranol is the most effective topical therapy for psoriasis. Dithranol prevents keratinocyte growth and the secretion of pro-inflammatory cytokines. When compared to the standard treatment of calcipotriol ointment, a randomized multicenter study involving 106 psoriasis patients with chronic psoriasis plaques showed significantly better therapeutic efficaciousness [38].

b. Indigo

An essential treatment in Traditional Chinese Medicine is “Indigo naturalis”. It is a blue powder made by grinding, fermenting, and adding lime to the plant *Baphicacanthus cusia* [36]. Numerous psoriasis trials have been conducted using indigo extract. Applying indigo extract twice daily for eight weeks proved to be effective in reducing psoriasis in 100 patients, based on a recently conducted double-blind, randomized, placebo-controlled trial. Both the 200 µg/g and 50 µg/g of indigo extract decreased the PASI by 70% and 50%, respectively. Some patients experienced local erythema, upper respiratory tract infections, and nasopharyngitis as side effects. Punch biopsies taken prior to and following an 8-week course of therapy demonstrated downregulation and a restoration of skin morphology [39].

c. Turmeric

In both Traditional Chinese Medicine and Ayurvedic medicine, turmeric is crucial. Turmeric, with its main active component, curcumin, has anti-inflammatory, antibacterial, and antioxidative qualities in vitro [40]. In recent years, a number of clinical and laboratory investigations have looked into curcumin’s potential as a treatment for psoriasis. Curcumin has the potential to alleviate psoriasis through its inhibition of phosphorylase kinase [41], reduction of pro-inflammatory cytokines like TNF-α and IL-17, and enhancement of the epidermis barrier through the in vitro induction of involucrin and filaggrin production [42].

d. Olibanum

Galen, Hippocrates, and Dioscorides all prescribed olibanum-containing ointments during the Greco–Roman era to treat a variety of skin conditions, including warts, psoriasis, bleeding, burns, and wounds. A total of 200 individuals with intermediate to mild psoriasis underwent three daily doses of an olibanum ointment containing 5% of 3-O-Acetyl-11-keto-β-boswellic acid for a period of 12 weeks in an open-label application study. The PASI dropped dramatically [36,43].

### 3.3. Herpes Simplex

Almost any area of the skin can develop blisters and sores due to the virus that causes herpes simplex. These sores typically appear on the genitalia and buttocks or around the mouth and nose. Numerous extracts, such as licorice extract and *Boswellia serrata* oleo gum, were reported to have antiviral action [44,45]. Clinical research utilizing these plant-based products is still lacking [36].

- Lemon balm

In a randomized, double-blind, placebo-controlled study, 66 individuals who had persistent herpes simplex labialis were evaluated with lemon balm cream. For five days, the test cream—a 1% dried *Melissa officinalis* extract—was applied four times a day. Lemon balm cream caused the lesions to resolve far more quickly, and the patients experienced less pain and blistering [46].

### 3.4. Wound Healing

Wound healing is an intricate interplay between the vascular system, cytokines, and various cell types, such as keratin cells, fibroblasts, and immune cells, that stop bleeding, eliminate pathogens, and initiate the process of re-epithelialization. Most traditionally used herbal remedies for wound healing have not been investigated as part of closely supervised clinical research [47].

- Birch bark

The molecular elucidation of betulin’s wound-healing characteristics demonstrates a positive impact on all three stages of wound healing [48]. In a split-thickness wound examination, the first clinical evidence for betulin’s wound healing properties was achieved through the topical application of a water-free betulin oleogel [49]. Following that, betulin oleogel was used in a number of multicentric, controlled, randomized clinical trials on second-degree burns and superficial wounds [50,51].

Similarly, other plants such as *Psidium guajava* and *Eucalyptus camaldulensis* also possess wound healing properties [52,53].

b. *Allium cepa* (Onion)

An onion extract was tested for its effect on scar formation in 58 participants who had minor skin surgeries, such as punch biopsies or the removal of skin tumors, in a randomized placebo-controlled study. Following three weeks of primary wound healing, the patients were given either onion extract or a placebo twice a day for ten weeks. When compared to a placebo, onion extract dramatically improved the scars’ overall appearance, smoothness, texture, and redness [54].

### 3.5. Acne Vulgaris

Perifollicular inflammation, the hyperproliferation of the epidermis, and hyperactive sebaceous glands are the hallmarks of acne vulgaris [36].

- Green tea

It may be demonstrated that the main polyphenol in green tea, epigallocatechin-3-gallate, has anti-inflammatory, sebosuppressive, and apoptotic properties on human sebocytes. Additionally, it exhibits antimicrobial properties against Propionibacterium acnes. An eight-week randomized split-face clinical experiment including 35 patients receiving either 5% or 1% epigallocatechin-3-gallate solution two times daily showed considerable improvement in acne [55].

b. *Melaleuca alternifolia* (Tea tree)

A randomized double-blind vehicle-controlled study comprising sixty patients with acne verified the effectiveness of a gel that included five percent tea tree oil when administered twice a day for forty-five days [56].

c. *Humulus lupulus* (Hop)

Hop extract has anti-inflammatory and antioxidant properties. Furthermore, in the agar diffusion test, a gel formulation with 0.3% *w*/*w* hop extract exhibited antibacterial activity against *Staphylococcus aureus* and *Propionibacterium acnes*. Thus, hop extract needs to be investigated in clinical trials as a potential alternative treatment for skin prone to acne [36,57].

### 3.6. Skin Cancer

Skin cancers, being the most prevalent cancers, are identified in Caucasians globally, and as a result of increased exposure to ultraviolet radiation, their incidence is steadily rising. Skin cancer is defined by an imbalance that favors either excessive cell survival and proliferation in the epidermis or insufficient apoptosis [58,59].

- *Panax ginseng*

In this study, red ginseng extracts applied topically were shown to suppress chemically produced skin cancers in mice [60].

b. *Rosmarinus officinalis* (Rosemary)

Extracts from rosemary (*Rosmarinus officinalis*) are said to possess antioxidant properties. The effects of a leaf methanol extract on mouse skin cancers were assessed. In mice given recognized chemical carcinogens, it was discovered that topically applied rosemary reduced the formation and growth of skin cancers. It seems that multiple extract constituents play a significant role in this process, even if the precise mechanism of action is currently being investigated. According to this research, antioxidant qualities were not the only thing that helped prevent skin cancers [61,62]. Some other plants used in skin disorders are listed in Table 1.

## 4. Traditional Treatment vs. Nanotechnology-Based Treatment

Nanotechnology uses nanoscale particles, nanofiber, and nanodevices to deliver drugs and other substances to specific cells in the human body. This is done for the purpose of treating diseases or injuries within the targeted cells and minimizing damage to other cells. Nanoparticulate medications have a number of benefits over traditional drug formulations, including improved bioavailability, a quicker onset of action, dosage homogeneity, and less variability between fasting and feeding, as discussed in Table 2. Nanomedicine has brought advancement in drug delivery and discovery.

Research and development advances are reflected in nanotechnology, which enhances product efficacy. In order to get around some of the drawbacks of conventional goods, nanotechnology is being used more and more in the cosmeceutical industry. Nano cosmeceuticals are now widely utilized to treat a variety of skin problems, including wrinkles, acne, photoaging, hyperpigmentation, etc. [72]. Lohani et al. recently analyzed a few nanoproducts made by a limited number of companies, such as Skin Caviar Ampoules, Hydra Zen Cream, and Revitalift, which use different nanotechnologies to manufacture phytocompounds. Vegetable oils and curcumin, two phyto bioactive substances that are nanosized, improve the appearance of skin through a variety of antioxidative processes. The products described above guard against premature aging and oxidative stress-induced skin aging [73]. Herbal cosmeceuticals are used to maintain the health and hygienic conditions of the skin. These products improve the skin’s condition and revitalize it at the cellular and molecular levels. The daily use of cosmeceuticals for skin care includes antioxidants, anti-inflammatories, skin rejuvenation agents, etc. [72]. Recent studies depict that phyto-based nano cosmeceuticals, which have improved protection, aesthetic, and health benefits, will certainly play an increasingly multifunctional role in the near future.

**Figure 2:**
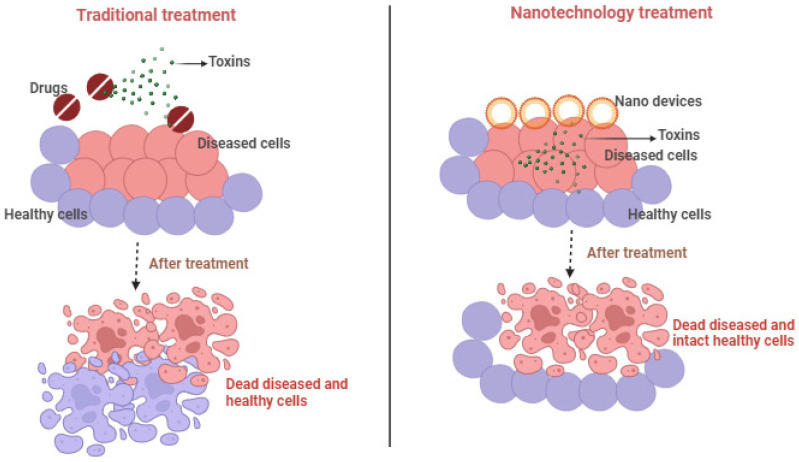
Comparison between traditional and nanotechnology-based treatment [75].

Herbal medicines have been used since ancient times for the treatment of various diseases, including skin diseases, but they also cause damage to normal cells as they do not provide targeted delivery. The nanotechnology-based delivery of herbal drugs provides targeted delivery and does not cause any harm to the uninfected or normal cells of the body [74]. The comparison between traditional and nanotechnology-based treatment on the basis of targeting is depicted in Figure 2.

## 5. Herbal Nanotechnology

The use of nanotechnology in combination with herbal extracts has been extensively documented in the literature due to the potential for nanostructured systems to increase the benefits of plant-based extracts, enhance the prolonged absorption of active ingredients, lower dosage requirements, lessen adverse effects, and enhance efficacy [76,77]. Many nanotechnological methods are being employed, such as liposomes, liquid crystal systems, polymeric nanoparticles, and precursor systems for liquid crystals [78]. These techniques enable the use of materials with various properties in a single formulation and may even alter a material’s properties and behavior in a biological environment. The delivery of drugs has been transformed by these technical advancements [79].

**Figure 3:**
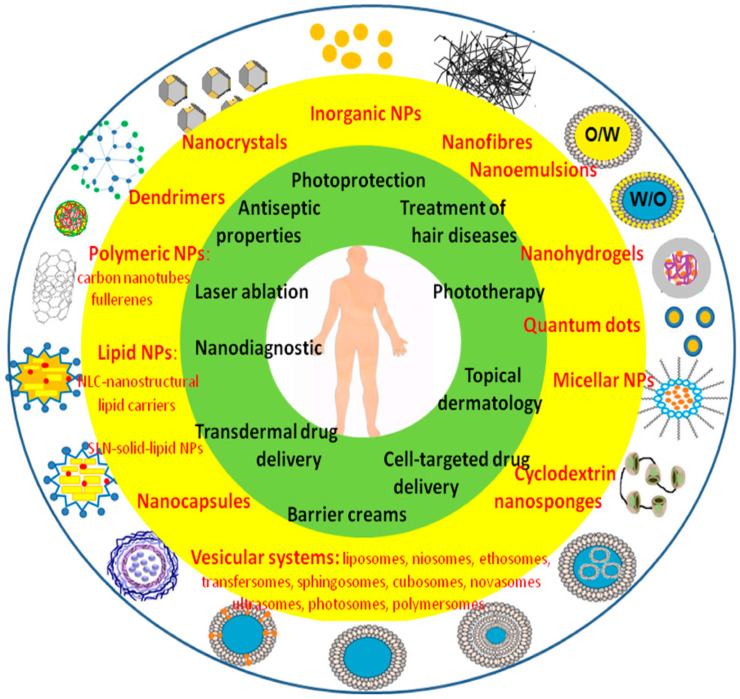
Various nanotechnology-based delivery systems and their uses for topical dermatological therapy. Adapted with permission from ref. [82] under Creative Commons Attribution License 4.0 (https://creativecommons.org/licenses/by/4.0/).

The ability to reintroduce inactive substances that were previously removed because they were inappropriate for formulation, in addition to increasing the strength of the active ingredients, is provided by the new drug delivery techniques [80]. The ability to improve novel compounds prior to their commercial release or medical application makes this approach even more alluring. Raising selectivity as well as efficacy, protecting against heat and photodegradation, reducing negative effects, and controlling the diffusion of active ingredients are a few instances of such improvements [81]. Various nanotechnological systems and their use are depicted in Figure 3 [82].

There is a need for improvements in nanotechnology and nanoscience, which pertain to the utilization of nanoscale materials, which, to date, have only been the focus of the cosmetics sector, along with advancements in recent decades connected to medical research. Scientific innovations can transform and improve approaches to solve challenging formulation preparation issues [83]. Nanostructures can successfully combine active ingredients with varying degrees of hydrophilicity and lipophilicity, as well as enhance the solubility and stability of active ingredients. The movement of a chemical to particular tissues or organs can also be targeted using this method [84].

To boost the absorption of the active ingredients, Bhattacharya and Ghosh utilized lipid-based systems and added green tea and ginseng (Araliaceae) extracts to several formulations [85]. Artemisia arborescens was used by Sinico et al. to create liposomes which showed that the technique assisted the active ingredients (β-thyjon and camphor, derivatives of azulene) from the plant in crossing the cytoplasmic viral barrier [86]. *Ocimum sanctum* L. (Lamiaceae) methanolic extract was used to create nanoparticles by Rajendran et al. They reported that when examined against *Bacillus subtilis*, *Escherichia coli*, *Pseudomonas aeruginosa*, and *Staphylococcus aureus*, the antibacterial activity of the encapsulated extract was superior to that of the free-form formulation [77]. Skin soft-tissue infections and scars can be efficiently treated using *Rhodomyrtus tomentosa* leaf-loaded transferosomes, which are natural biomedicines [87,88]. Similarly, other nano formulations such as phytosome-loaded shape memory gels for skin aging [89] and the microemulsion of the oil of *Kaempferia galanga* for UV ray protection [90] have been formulated. It is an intriguing strategy to enhance a formulation’s most appealing characteristics by using various drug delivery systems that utilize nanotechnology. Additionally, nanoscale particles might be an indication of the time when activity is guaranteed and issues with employing medicinal plants are resolved [14].

The different herbal formulations based on different nanotechnology-based systems are as follows.

### 5.1. Polymeric Nanoparticles

The harungana of Madagascar Lam. Ex Poir is well renowned for its ability to fight off viruses, fungi, and bacteria. The antibacterial efficacy of an ethanol-based extract of HLE (*Harungana madagascariensis*) leaf combined with poly (D, L-lactide-co-glycolide) nanoparticles, i.e., PLG-NPs, was assessed and compared by Moulari et al. Two Gram-positive strains of S. epidermidis and Micrococcus luteus, as well as a Gram-negative strain of Moraxella species, were evaluated ex vivo against one concentration of HLE, while two concentrations of HLE—500 g/mL and 1000 g/mL—were taken into consideration for the in vivo experiment. An artificial contamination technique was used to determine the ex vivo antibacterial characteristics of *S. epidermidis* CIP 55109. The bacterium was injected into the human skin surface for 12 h. Studies conducted in vitro demonstrated that both formulations totally inhibited the growth of all tested bacterial strains. Four hours after artificial contamination, ex vivo testing revealed that the HLE-PLG-NPs had more antibacterial properties than the HLE solution. Incorporating extracts into polymeric nanoparticles led to better results. The thin-layer chromatography study showed peaks of only two compounds in the case of nanoparticles, demonstrating greater efficacy by loading in the form of a nanocarrier system. The main anti-bacterial activity was due to presence of the flavonoid heteroside [14,91].

Sun and coworkers examined the aqueous solubility, chemical stability, resolution of the epidermal barrier, and in vivo anti-psoriatic activity of curcumin in PLGA (polylactic-co-glycolic acid) nanoparticles. The results show that PLGA nanoparticles distribute and safeguard curcumin during the delivery procedure and enable it to reach the dermis. Therapeutic benefits are influenced by sustained medication release and the compatibility of skin and skin layer penetration. Better therapeutic benefits are demonstrated with the incorporation of curcumin into PLGA nanoparticles, showing sustained medication release, compatibility of the skin, and skin layer penetration [92]. In order to target heat shock protein 70-1, Raghuwanshi and coworkers employed the flower extract of *Woodfordia fructicosa* heat shock protein 70-1, which has the potential to be inhibited in the therapy of psoriasis. The authors developed *Woodfordia fructicosa* extract-based biogenic gold nanoparticles containing ellagic acid, quercetin, and myricetin as the main therapeutic components and came to the conclusion that the resulting biologically produced nanostructured formulation would be an effective substitute for treating psoriasis. The results depicted the targeted release of nanoparticles as compared to conventional formulation [93,94].

Lee et al. created an siRNA delivery system based on poly lactic-co-glycolic acid nanoparticles and paired it with a functional laser to enhance the absorption of skin for topical psoriasis treatment. The ability of the nanocarriers to reduce IL-6 expression was demonstrated by their low cytotoxicity and simple cellular absorption. The nanoformulation that included a cationic nature surfactant for ion coupling with siRNA was successful in knocking down IL-6 in keratinocytes and macrophages with 66% and 77% efficiency, respectively. The lasers improved the naked siRNA’s permeability by 3.7–5.0 times. The combination of the laser and nanosystem reduced epidermal hyperplasia and macrophage infiltration, according to the histological analysis. Naked siRNA distribution that was passive or aided by laser had less success at reducing dermatitis. In mice, a topical administration of fractional laser-assisted nanoparticles caused a 56% decrease in IL-6. The study depicts that the efficacy of SiRNA increased when loaded into nanoparticles, but were found to show a cytotoxic effect in the naked form [95].

Lin et al. developed nanocarriers of polylactic-co-glycolic acid containing dictamnine. The results showed that the nanocarrier system penetrated the dermal layer more effectively than bare dictamnine, and it also reduced in vivo inflammatory cytokine expression and the symptoms of dermatitis. The formulation enhanced the anti-inflammatory potential of dictamnine by providing prolonged release with greater penetration [96]. Other research-based evidence for herbal nanoformulation using polymeric nanoparticles is summarized in Table 3.

### 5.2. Solid Lipid Nanoparticles Nanostructured Lipid Carriers (NLCs)

In order to assess the formulation’s viability as a dermal delivery system, Guo et al. added quercetin to NLCs (QU-NLCs). The formulation was created utilizing the method of emulsion evaporation–solidification at lower temperatures and includes quercetin, stearic acid, glyceryl monostearate, and soy lecithin. The mean size of the nanoparticles was 215.2 nm, and their average entrapment effectiveness was 89.95% ± 0.16%. They were spherically formed. As a result, the inclusion was successful in promoting quercetin penetration, increasing the amount of quercetin maintained in the dermis and epidermis, and enhancing the flavonoid’s anti-inflammatory and antioxidant activities. Further evidence from this study showed that NLCs have excellent dermal delivery potential, targeting capability, and a delayed release [14,104].

Agrawal et al. formulated nanostructured lipid carriers of capsaicin derived from *Capsicum annum* for treating psoriasis. In the case of psoriasis, NLCs exhibit increased skin permeation through tough and hyperproliferative skin. Since no skin irritation symptoms have been noticed, the scientists hypothesized that both types of lipid nanoparticles would be suitable for cutaneous administration [105,106].

Recently, Montenegro and colleagues suggested using nano-encapsulated rosemary essential oil to increase skin hydration and elasticity. After being loaded into lipid nanoparticles and added to Carbopol hydrophilic gels, an essential oil with well-known antibacterial, antioxidant, wound healing, and hydration capabilities was used on human volunteers. Cetyl palmitate served as the solid component of the nanoparticles, and rosemary essential oil served as the liquid component, creating nanostructured lipid carriers as a result. Skin hydration and elasticity changes were seen after the application of rosemary essential oil combined with nanostructured gel for a week. In comparison to ordinary gels, nanostructured gels caused better skin hydration and elasticity. In the event of skin dehydration, the researchers have proposed the use of a nanostructured gel containing rosemary essential oil [107].

The effectiveness of NLC containing thymol as a topical vehicle for the treatment of skin inflammation and wound healing has been examined by Pivetta and colleagues. Since the produced gel revealed an anti-inflammatory effect in two different animal models, it proved suitable for the management of inflammatory skin conditions. Additionally, the imiquimod-induced psoriasis mouse model’s healing was enhanced by the thymol-containing nanostructured gel [108].

Eugenol-loaded SLN was created, manufactured, and defined by Garg and Singh. The integration of SLN into a Carbopol hydrogel produced a nanostructured vehicle that may be used to administer SLN topically. In comparison to a medicinal oil solution, a greater eugenol accumulation was seen in the epidermis. In addition, compared to an ordinary gel or untreated skin, an occlusion investigation showed that human cadaver skin was more hydrated after the application of the nanostructured gel. The scientists have proposed using a nanostructured gel that contains eugenol for the treatment of cutaneous fungal infections [109,110].

The different studies performed by researchers using NLCs or SLNs of herbal drugs showed that the NLC or SLN preparations had better potential than their traditional formulations. Moreover, it can be concluded that formulating NLCs or SLNs and loading them into gel could provide enhanced penetration, prolonged release, and better efficacy.

### 5.3. Ethosomes

The ethosomes of curcumin from *Curcuma longa* were formulated in a study using hyaluronic acid for treating psoriasis. The results demonstrated a decrease in drug leakage as well as an improved penetration and retention of curcumin. Similarly, ethosomes of thymoquinone, an anti-psoriatic molecule derived from *Nigella sativa*, showed positive outcomes for psoriasis treatment with enhanced solubility and penetration [106,111]. In a study by Sun and coworkers, which was discussed in the section on polymeric nanoparticles, the curcumin nanoparticles enhanced penetration and provided sustained release, while the formulation of ethosomes of curcumin demonstrated enhanced solubility and decreased leakage of the drug along with greater and prolonged penetration.

In order to obtain lycopene-rich extracts from tomatoes, Ascenso and colleagues developed their dispersion. As bilayer softeners, the dispersions contained Tween 80 with soybean phosphatidylcholine for transferosomes and ethanol for the ethosomes. In particular, the skin permeation and retention investigation showed that ethosomes containing lycopene were retained in the skin for a longer time, whereas confocal microscopy images showed that transferosomes containing fluorescent rhodamine were taken up by HaCat cells. Lycopene-containing vesicles were applied to an ear edema model induced by anthralin to examine their ability to penetrate inflamed skin. In comparison to the simple lycopene extract, transferosomes and ethosomes were both able to lower the levels of inflammatory cell infiltration and epidermal hyperplasia. Vesicles possibly improved lycopene retention in the skin by enhancing carrier-mediated lycopene skin delivery, which probably improved skin retention and increased the therapeutic impact [110,112].

Vitamins A, E, and C were used in conjunction to create ethosomes by Koli and Lin. Due to the synergistic effects of all vitamins, as well as their distribution to the innermost layers of the skin, the prepared formulation demonstrated stronger antioxidant properties in comparison to the usual drug delivery system [113,114].

### 5.4. Liposomes

Lin et al. investigated camptothecin-containing liposomes coupled to α-melanocyte-stimulating hormone (α-MSH) to preferentially target melanoma cells. The camptothecin release may be managed by the liposomes, which were based on stearylamine, phosphatidylcholine, and cholesterol. In comparison to non-targeted liposomes and free camptothecin, greater cell endocytosis was seen in the α-MSH liposomes using fluorescent microscopy. Notably, the scientists showed that α-MSH liposomes were primarily internalized in the cytoplasm. The capacity of α-MSH liposomes to target tumors showed that they may be able to increase the effectiveness of camptothecin against melanoma [110,115]. The study depicts the targeted release of camptothecin-loaded liposomes providing better efficacy against melanoma as compared to the non-targeted formulation.

Using a murine model, researchers examined the anti-inflammation characteristics of liposomes intended for the topical application of curcumin and quercetin. They found that adding penetration enhancers to liposomes greatly increased these characteristics while also postponing the development of TPA-induced (12-O-tetradecanoylphorbol-13-acetate) wounds [114,116].

**Figure 4:**
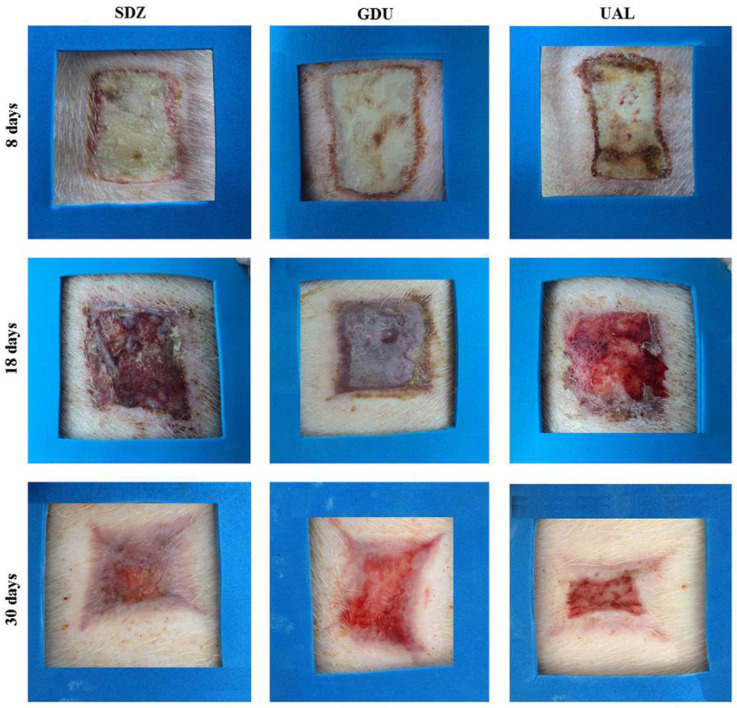
Macroscopy of the wound treatment at 8, 18, and 30 days with silver sulfadiazine (SDZ), DuoDerme^®^ (GDU), and a gelatin layer loaded with usnic acid liposomes (UAL). Adapted with permission from [117].

The evaluation of the usnic acid-loaded liposomes in gelatin by Rabelo et al., led to favorable findings for the treatment of wounds. According to these findings, the liposome membrane plays a significant role in controlling second-grade infections in the pig model. Additionally, the liposomal membrane-treated group showed deposition on cellularized granulation tissue, which, in contrast to one of the commercial products, increased the maturation of granulation tissue and mended the wounds (Figure 4) [117].

The above two studies done by researchers for the treatment of wounds demonstrate that the application of nanotechnology in the formulation increased their therapeutic effect and penetration and also prevented the development of other induced wounds in the future.

### 5.5. Nanoemulsions

Mahdi et al. created nanoemulsions that included *Phyllanthus urinaria* extract using esters made from palm kernel oil. These showed DPPH radical neutralizing action, the neutralization of reactive oxygen species, and the prevention of oxidative damage brought on by UV light when they were tested for their anti-aging effects due to the presence of polyphenols in the extract (gallic acid, geraniin, and ellagic acid) [114,118]. The formulation of nano emulsion-based cream increased the antioxidant potential activity along with permeation, providing a better anti-aging effect. The partitioning of the drug into two phases enhanced the solubility and drug release.

Vater et al. formulated nano emulsions of spruce balm and birch bark extract using lecithin for wound healing properties. The nanoemulsions loaded with either birch bark or spruce balm extract resulted in greater fibroblast and keratinocyte cell viability rates. After the treatment, there was increased keratinocyte and fibroblast proliferative activity, which is necessary for wound repair. Our research suggests that nanoemulsions of herbal extracts are remarkable wound-healing medications, and their use in lecithin-based nanoemulsions may be a useful wound-care therapy [119]. Kreutz et al. formulated a hydroxyethyl cellulose hydrogel of the nanoemulsion of *Aniba canelilla* to evaluate its anti-inflammatory activity on the skin. *Aniba canelilla* essential oil-containing hydroxyethyl cellulose-hydrogel thickened nano emulsion appeared to be an effective formulation because it demonstrated a clear anti-inflammatory potential with decreased myeloperoxidase activity. It acted by reducing polymorphonuclear leukocyte movement, decreasing edema and reducing the release of inflammatory mediators. 1-nitro-2-phenylethane and methyleugenol were found to be the components of essential oils responsible for activity [120].

**Figure 5:**
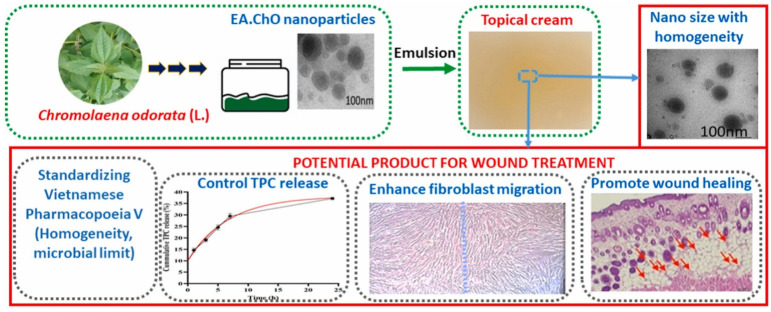
Nanoparticle-loaded cream of *Chromolaena odorata* for the treatment of wounds by controlling TPC (total phenolic compound) release, enhancing fibroblast migration. Adapted with permission from [121].

Luu et al. through the use of an oil-in-water emulsion, formulated a topical cream based on a nanoformulation of *Chromolaena odorata* leaves with a fraction of ethyl acetate extract. The cream was formulated by loading active ingredients (flavonoids and tannins) in pluronic micelles. The formulated cream demonstrated high homogeneity, a sufficient pH level, the sustained release of phenolics, and satisfactory stability for prolonged storage. When DPPH was incubated with cream for 15 min to 5 h, the amount of free radical scavengers decreased from 10% to 80%. Additionally, the cream encouraged the movement of fibroblast cells through the appropriate release of phenolic chemicals, but the extract in the same quantity caused toxic effects and prevented wound closure [121]. The results of the formulated nano-emulsion-based topical cream are depicted in Figure 5.

Chlorogenic acid nanoemulsions were developed by Budama-klinic et al. for hyperpigmentation. The in vitro cell survival result, the Ames test result, and the formulation’s non-mutagenicity were used to demonstrate that the formulation was suitable for transdermal application. The finished chlorogenic acid nano emulsion formulation was found to decrease the activity of tyrosinase and melanogenesis during efficacy tests on melanoma B16 cells [122]. The therapeutic efficacy of chlorogenic acid is limited due to poor penetration, which was improved by formulating its nanoformulation, providing greater intradermal delivery. The prepared nanoemulsion increased the efficacy and safety of chlorogenic acid in hyperpigmentation. The other emulsion-based nanoformulations are detailed in Table 4.

### 5.6. Niosomes

In order to create a topical gel, Priprem et al. created niosomes that encapsulated a concentrate of *Zingiber cassumunar*. Improved chemical stability and skin permeability were seen. The anti-inflammatory effects due to the presence of active compound(*E*)-4-(3′,4′-dimethoxyphenyl) but-3-en-1-ol were comparable to the ones of commercially available hydrocortisone cream and Piroxicam gel [127]. It can be concluded that in comparison to steroids and other conventional gel formulations, the noisome-loaded gel of *Zingiber cassumunar* provided better stability, increased anti-inflammatory effect, and enhanced permeation.

In different vesicular forms, including ethosomes, liposomes, and transferosomes, an alcohol-based extract of *Curcuma longa* was produced. The most effective cream was the one filled with *Curcuma longa* transferosomal extract containing curcumin; it was followed, in order of effectiveness, by ethosomal, liposomal, and *Curcuma longa* extract creams. When these forms were coupled with the cream, the skin damaged by UV radiation showed improved recovery [114,128].

**Figure 6:**
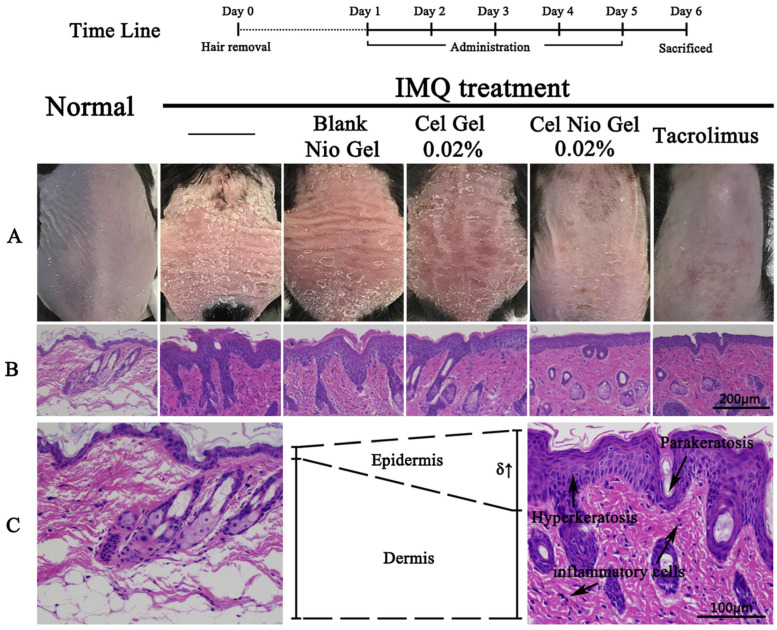
(**A**) Images of dorsal skin of mice after administration of Imiquimod (IMQ). (**B**) Staining with H&E demonstrating the changes in inner skin after the application of tacrolimus ointment, blank niosomal gel, and Celastrol gel (positive control). (**C**) An enlarged view of the IMQ-treated group (**right**) and normal (**left**) groups. Adapted with permission from [129].

Meng et al. created niosomes of Celastrol incorporating Span 20, 60, and cholesterol by a thin-film hydration method. For prolonging the effects of the topical medication on the skin and preserving subcutaneous hydration, the hydrogel was utilized as a core carrier. In comparison to the Celastrol hydrogel, the drug concentration of the Celastrol niosomal gel was shown to be about 13 times higher in the skin in an in vitro penetration assay (Figure 6). Erythema and scaling on the lateral skin were further improved [129].

### 5.7. Nanofibers

Nanofibers operate as a sheet that enhances the tissues since they are made of indestructible chains of polymers of both synthetic and natural substances [130]. Emodin, an anthraquinone compound that can be found in the rhizomes of *Rheum officinale* L., is widely used for wound healing because of its antibacterial and anti-inflammatory properties. When applied to severe skin injuries, it had a beneficial outcome [131]. In comparison to the pure substance, the emodin nanofibers in polyvinylpyrrolidone were non-toxic, anti-allergenic, bioactive, and disintegrated quickly. Re-epithelization was demonstrated to have taken place at the site of injury, accelerating the healing process [132]. Emodin was added to cellulose acetate nanostructure fibers to bring the collagen content of human cells to 100%. Through the incorporation of herbal components into cellulose acetate nanofibers, wound healing is accelerated by the use of biological nanomaterials [133]. Chitosan nanofibers that had been loaded with bromelain led to successful wound healing. Positive effects were seen for second-degree burns. When compared to chitosan 4% *w*/*v* bromelain, the chitosan 2% *w*/*v* bromelain produced better physiochemical outcomes and was successful in minimizing burn-induced damage [133,134]. The different nanofiber-based formulations of emodin discussed above show better outcomes than those of its pure form. The loading of emodin in nanofibers increased wound healing activity and decreased the associated side effects. Nanofiber-based herbal nanoformulations for skin diseases are listed in Table 5.

### 5.8. Hydrogels and Nanogels

**Figure 7:**
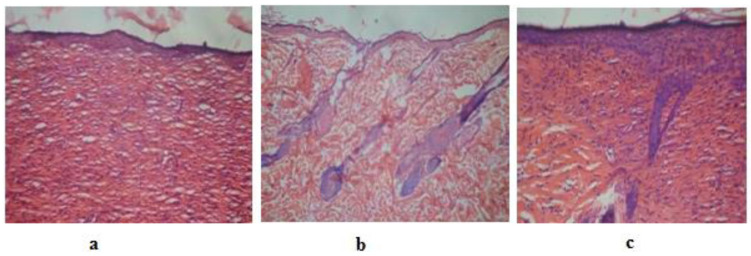
Illustration depicting images of animal skin: (**a**) untreated; (**b**) standard treatment; and (**c**) quercetin-loaded liposome treatment. Adapted with permission from [138].

Hydrogels made of sodium alginate and polyvinyl alcohol were created by Esposito et al. as a novel delivery mechanism, primarily to treat inflammation and skin aging. A hydrogel that was loaded with quercetin showed a respectable swelling and viscosity profile. The obtained outcomes demonstrated that a quercetin-loaded hydrogel reduces the rate of infiltration and increases the penetration and duration of the drug–skin interface, consequently enhancing the action of quercetin [137]. Jangde et al. used liposomes loaded with quercetin to construct a multiphase hydrogel system and assessed its structure, swelling index, water vapor transfer rate, stability evaluation, hemocompatibility, and in vitro and in vivo testing (Figure 7). The results showed that the hydrogel considerably accelerated the process of wound closure and enhanced wound healing applications [138].

Bagde et al. produced a topical nanogel loaded with quercetin and TiO_2_, and they used the Box–Behnken design to optimize the low and high concentrations of quercetin nanocrystals. Over 70% of the medication was released within 24 h using combined nanogels. The new combination of quercetin and titanium dioxide nanogel inhibits the inflammatory and cell cycle pathways, as demonstrated by the in vivo animal model. Overall findings, therefore, indicated that the proposed nanoformulation could enhance skin deposition and can be used as an innovative pharmaceutical delivery strategy against UVB-induced skin cancer [139]. The different formulations of quercetin formulated by researchers, either as a hydrogel or nanogel, show their improved efficacy in the treatment of skin problems. The formulation by Jangde et al. depicts increased bioavailability along with faster wound healing in comparison to other formulations. The other nanogel-based herbal nanoformulations are summarized in Table 6.

### 5.9. Others

The nanocomplexes were created by Nirmal et al. by loading gold nanorods and isatin into a polylactic-co-glycolic acid matrix. This combination has been found to have synergistic anti-psoriatic activity. The combined nanocomplexes with near-infrared prevented epidermal hyperplasia and neutrophil infiltration, according to the in vivo psoriasis murine model. After photothermal treatment, the elevated cytokines in the area of injury could return to their baseline levels. For at least five days, the subcutaneous nanocomplexes persisted in the skin. The skin and liver of normal mice had no harm from the nanocomposites [143]. A 6-Gingerol-loaded self-nano emulsifying drug delivery system as a nano emulgel was developed by Ahmad et al. in order to enhance topical administration through improved solubility and skin penetration for the control of wound healing and anti-inflammatory processes. In order to improve skin permeability, a nano emulgel was utilized, and the dermatokinetic results revealed a substantial improvement in the treated skin as compared to a conventional gel. It increased skin permeability and solubility. The topical application of the nano emulgel demonstrated improved wound healing and anti-inflammatory effects [144].

Amer et al. formulated aspasomes (quercetin loaded in nanovesicles of vitamin C) for the treatment of acne. Quercetin’s antioxidant activity was preserved by aspasomes, which also demonstrated a markedly greater antibacterial impact towards Propionibacterium than quercetin alone and was safe on fibroblastic cells. In the clinical study of 20 acne patients, quercetin aspasomes showed decreased percentages of 77.9%, 11.8%, and 55.3% for inflammatory lesions, comedones, and total lesions, respectively [145].

Other nanoformulations for the treatment of various skin diseases are outlined in Table 7.

The different studies discussed above show the potential of herbal nano formulations in treating skin diseases and the increased popularity of herbal products. The application of nanotechnology in the development of various formulations will help in effectively curing various life-threatening skin disorders by providing targeted and prolonged effect with decreased side effects.

## 6. Patents

The different patents published on herbal nanoformulations for the treatment of skin diseases in the last 15 years are discussed in Table 8.

## 7. Current Challenges and Future Perspectives

Herbal medicine delivery systems using nanoscale technology may enhance biological activity and address problems with plant-based pharmaceuticals. Yet, there are still numerous challenges in this discipline to overcome before implementing clinically sound treatments. One of the biggest challenges in converting this technology into medicines is testing novel ways to control how nanomaterials interact with biological systems. The potential for obtaining multifunctional systems to satisfy various biological and therapeutic requirements, as well as the viability of the increased production of processes that quickly bring creative therapeutic technology to market, are novel obstacles in the design of nanotechnology-based systems for drug delivery. Investigating the targeting effectiveness of nanoparticles and meeting global criteria for their toxicological and biocompatibility are some additional emerging issues. A better method of delivering medications to the target site at a dosage that does not modify the way the disease is currently being treated must be developed. It is obvious that more studies are required to determine the effectiveness, safety, and ideal applications of herbal treatments and establish standards for them. Researchers also need to concentrate on creating biocompatible and biodegradable nanomaterials. Continued research and successful pre-clinical and clinical studies are required to provide better alternatives to conventional formulations. Along with this, the contribution of health professionals and the general public is necessary to increase the acceptance of herbal nanoformulations for various skin diseases all over the world.

## 8. Conclusions

The data compiled in this research have shown that treating skin diseases with herbal medicines based on nanotechnology is a novel approach. In fact, there are many clinical advantages of applying herbal medicines via nanotechnology systems to the skin. The ability to use a formulation that is entirely biocompatible guarantees the consumer a “green” approach and virtually zero side effects when administered topically. Several nanotechnology-based approaches, such as liposomes and solid lipid nanoparticles, have been recommended for topical administration, indicating their potential in inflammatory phenomena, wound healing, fungus infections, and skin aging. In general, the various investigations presented here have indicated that the topical application of nanosystems provides a longer release and efficacy of the loaded herbal compounds. Regardless, a lot more research needs to be done and addressed to understand more about the interaction of nanosystems with the skin.

## Figures and Tables

**Table 1:** Plants used for skin diseases.

Name of Plant	Part of Plant	Use	Research	Ref.
*Oryza sativa*	Seeds	Antioxidant and anti-inflammatory activity, antibacterial effect, antifungal effect, psoriasis, and anti-aging agent	Palungwachira et al. evaluated the cellular activity of anthocyanins, which will be useful in the creation of new products that promote natural healing. Based on the modulation of type-I collagen gene expression and the suppression of H_2_O_2_-induced activation of necrosis factor-B in skin fibroblasts, the data indicated that anthocyanins from *Oryza sativa* have anti-inflammatory and anti-aging potential.	[63]
*Nicotiana tabacum*	Leaves	Antibacterial, antifungal, wound healing, and anti-aphthous activity	Sharma et al. used a Wistar rat model to examine the wound-healing properties of an ethanolic extract of stems of *Nicotiana tabacum*. Studies conducted in vitro and in vivo have shown the high efficiency of ethanolic extracts in wound healing, suggesting that they could be applied topically as herbal medicine.	[64]
*Knema globularia*	Seeds, leaves	Cytotoxic activity and antioxidant activity	Thinh et al. determined the antioxidant effect of the leaves of *Knema globularia*. The findings demonstrated the potent antioxidant qualities of the methanol essential oil of *Knema globularia*, suggesting that they may offer a novel and dependable supply of naturally occurring antioxidants.	[65]
*Senna macranthera*	Flowers	Antifungal activity	Nascimento et al. evaluated the antifungal and cytotoxic effects of flowers of senna species. The current findings indicated that flowers of *Senna macranthera* are a promising source of novel antifungal compounds.	[66]
*Punica granatum*	Pericarp	Anti-melanoma, anti-inflammatory, antibacterial, and wound healing	Saini et al. utilized the peel extract of *Punica granatum* and evaluated its antibacterial activity against multidrug-resistant bacteria. Strong antibacterial activity was observed in the interaction with *Punica granatum* peel extract, with a minimum inhibitory concentration of 780–6250 μg/mL and a zone of inhibition measuring 24.33 mm.	[67]
*Aloe vera*	Leaves	Insect bites, sunburns, wound infections, scabies, wound healing, burns, itching, and swelling [19]	Padmakar et al. evaluated natural aloe vera’s safety and effectiveness in treating stable vitiligo. It was found that using natural aloe vera to treat stable vitiligo was both effective and safe. However, in order to confirm the effectiveness and safety of *Aloe vera* in the management of vitiligo, higher sample sizes for randomized controlled trials are advised.	[68]
*Jatropha curcas*	Seeds, roots, leaves	Wound healing and skin lesions [19]	An herbal ointment with pro-wound healing properties was developed and tested, incorporating the methanol leaf extract of *Jatropha curcas*. The application of the ointment batch containing the maximum amount of *Jatropha curcas* extract showed the highest rate of wound closure in comparison to the blank ointment, which had an epithelialization duration of 18.8 days.	[69]
*Glycyrrhiza glabra*	Root	Allergic dermatitis, depigmentation, and atopic dermatitis [19]	Jan et al. isolated different polyphenols from the extract of *Glycyrrhiza glabra* and found flavonoids and other compounds showing antioxidant activity.	[70]
*Ficus carica*	Fruit	Skin ulcers, acne, hyperpigmentation, eczema [19]	Khan et al. sought to determine the effect of cream containing fruit extract from *Ficus carica* on skin parameters such as erythema, sebum, moisture content, trans-epidermal water loss, and skin pigmentation. The pigment melanin, trans-epidermal water loss, and skin sebum were all considerably reduced by the formulation and had a negligible impact on skin erythema while greatly increasing skin moisture.	[71]

**Table 2:** Traditional vs. nanotechnological approach.

Features	Traditional Herbal Formulation	Nanotechnological Herbal Formulation
Particle Size	Larger	Nano-size
Solubility	Limited Solubility	Higher solubility
Bioavailability	Limited Bioavailability	Higher bioavailability due to small size
Targeted Delivery	No	Provides targeted delivery to specific cells
Dosing Precision	Less precise	More precise
Surface Area	Less surface area for interaction	More surface area for interaction
Production Process	Simple	Complex
Allergic Reactions	More allergic reactions to extract	Less, as the drug is enclosed in nanoformulation
Interaction with other drugs	Possible	It may or may not be possible
Skin Irritation	Yes	No
Efficacy	Low	High

**Table 3:** Herbal formulations based on polymeric nanoparticles for skin diseases.

Plants	Approaches	Techniques	Skin Disease	Types of Study	Outcomes	Ref.
*Thespesia populnea*	Nanoparticles	Aqueous and methanolic extraction methods	Skin infection	UV–vis spectroscopic analysis Scanning electron microscopy Energy-dispersive X-ray spectroscopy Fourier-transform infrared spectral analysis (FTIR) Zeta potential Antimicrobial activity by disc diffusion method	Antimicrobial activity of nanoparticles was more effective against skin infection Zeta potential value of 3.50 mV indicated the negative charge Treatment of skin infection Wound healing process	[97]
*Alpinia calcarata* (diterpenoids, flavonoids and phenols)	Nanoparticles	Aqueous and methanolic extraction methods	Skin cancer	UV–vis spectroscopy SEM EDAX PXRD FTIR	Treatment of infections and skin cancer Enhanced skin permeation Sustained release Great physicochemical stability Synergistic effects Dose-dependent reduction	[98]
*Fumaria officinalis* (stylopine, sanguinarine)	Nanoparticles	Ionotropic gelation method	Wound healing	Microarchitecture Cell toxicity Cell mobility activity Cell protective potential Anti-inflammatory activity Drug release rate In vivo study	Migration activity The animal had noticeably higher healing of wounds, epithelium thickness, and deposition of collagen Treating diabetic wounds Showed anti-inflammatory effects and therapeutic benefits	[99]
*Praecitrullus fistulosus*	Silver nanoparticles	Solvent casting method	Wound healing	Ultraviolet-visible (UV–vis) spectroscopy High-resolution X-ray diffractometer High-resolution transmission electron microscopy	Higher water absorption capacity Higher folding endurance and porosity Improved surface roughness Thermal stability Higher antimicrobial activity	[100]
*Moringa oleifera* (phenolic compounds and flavonoids)	ZnO nanoparticles	Sonicate method	Anti-acne	UV–vis spectrum Fourier-transform infrared spectroscopy X-ray diffraction analysis Scanning electron microscope analysis Anti-acne efficacy Antibacterial efficacy	Higher antioxidant activity having a half-maximal inhibitory concentration Anti-acne activity Enhanced antibacterial activity	[101]
*Fragaria ananassa* (flavonols, flavan-3-ols, anthocyanins, hydrolyzable tannins)	Copper nanoparticles	----	Skin wounds	FTIR, UV spectroscopy SEM TEM Microbiological study In vivo design	Anti-inflammatory Anti-ulcer Astringent Anti-allergic Antibacterial Antifungal and antidiarrheal activities CuNPs showed higher antibacterial and antifungal properties Antifungal properties	[102]
*Ximenia americana* L. (rutin, epicatechin, catechin, myricetin)	Silver nanoparticles	Solvent casting method	Wound healing	In vivo study. In vitro study	Exhibited increased levels of skin resistance Reduced the number of neutrophils and macrophages Rise in collagen fibers and fibroblasts Reduced the inflammatory process Enhanced collagen fiber deposition	[103]

**Table 4:** Herbal formulations based on nanoemulsions for skin diseases.

Plants	Approaches	Techniques	Skin Disease	Types of Study	Outcomes	Ref.
Garlic (dialkyl polysulphides) and ginger (zingiberene, curcumene, and β-bisabolene)	Nanoemulsion	Ultrasonic cavitation	Skin wound healing	Droplet size zeta potential Refractive index viscosity transmittance FT-IR HPLC Antimicrobial studies (in vitro) Stability studies	Quick recovery, with 86% to 100% of the wound healed in just 9 days Showed anti-inflammatory activity Enhanced wound healing potential and promoted fast epithelization	[123]
*Chromolaena odorata* *(flavanoids, tannins)*	Oil-in-water emulsion Pluronic micelles	-----	Burn wound healing	Organoleptic properties In vitro release study Stability studies	Good homogeneity Good stability Suitable release Enhanced the effectiveness of wound healing	[121]
Chlorogenic acid	Nanoemulsion	Ultrasonic homogenization method.	Hyperpigmentation disorder	Genotoxicity/mutagenicity and cytotoxicity tests In vitro and in silico analysis Molecular docking study Pharmacokinetic analyses	Kinetic and thermodynamic stability Skin-lightening effects Higher efficacy Safety profile	[122]
*Aniba canelilla* (Kunth) (1-nitro-2-phenylethane and methyleugenol)	Nanoemulsion	----	Skin disorders	Droplet size Polydispersity index Zeta potential PH Transmission electron microscopy Release studies Skin permeation essays	Proven anti-inflammatory activity Controlled release Increased nanoemulsion viscosity and adhesiveness Myeloperoxidase activity and interleukins content	[120]
*Alpinia galanga* extract (1-acetoxychavicol acetate)	Nanoemulsion	----	Psoriasis	Mean droplet size Zeta potential Ex vivo permeation studies	Mean droplet size was found to be 60.81 ± 18.88 nm Zeta potential −7.99 ± 4.14 mv Exhibited a ten-fold rise in flux A decrease in the psoriasis area severity index	[124]
*Brosimum Gaudichaudii* *(furanocoumarins bergapten and psoralen)*	Microemulsions	----	Vitiligo	Stability studies In vitro skin permeation studies In vitro biological assays Irritant potential	Burn wounds repair Burn healing effect Physically stable	[125]
Linseed oil	Nanoemulsion	Ultrasonic emulsification method	Atopic dermatitis	In vitro Ames/salmonella assay Physicochemical stability tests Mean droplet size PDI Zeta potential	After 24 h, 78.4% of the formulation was released No mutagenic effect Kinetic and thermodynamic stability Optimal physicochemical characteristics and maximum stability	[126]

**Table 5:** Herbal formulations based on nanofibers for skin diseases.

Plants	Approaches	Techniques	Skin Disease	Types of Study	Outcomes	Ref.
*Malva sylvestris* (mucilage and flavonoids)	Nanofibers	Maceration method	Wound dressings	In vivo wound healing Assessment of swelling ratio Histomorphometry analysis Statistical analysis	Improve the absorption ability of wound exudates Antibacterial activity Effective at reducing both acute and long-term inflammations	[135]
*Ananas comosus* (bromelain)	Nanofibers	Electrospinning method	Wound healing	In vitro release study Enzymatic activity of bromelain Swelling test Loading analysis Cytotoxicity test In vivo studies	Burn wound repair Burn healing effect Induced burn wounds in rats Exhibited reduced cytotoxicity and improved physicochemical characteristics and release profile	[136]

**Table 6:** Herbal formulations based on nanogels for skin diseases.

Plants	Approaches	Techniques	Skin Disease	Types of Study	Outcomes	Ref.
*Calotropis procera* (flavones, tannins, and alkaloids)	Nanogel	Diffusion technique	Skin acne and skin cancer	Microbiological study UV spectroscopy FTIR SEM.	Showed antioxidant and anticancer activity Treatment of skin cancer and acne Synergistic action of the flower extract Exhibited strong antibacterial activity	[140]
Sesame oil (lignans and sesamol)	Hydrogel	---------	Skin hyperpigmentation	Size of particles Zeta potential Refractive index Electrical conductivity pH Stability	The hydrogel structure’s particle size was improved Prevent UV radiation-induced skin damage effectively	[141]
*Smilax china* and *Salix alba* (quercetin)	Nano lipid carrier-based gel	Sonication method	Psoriasis	Particle size Polydispersity High entrapment TEM Drug release Dermal transport studies Thermo-analytical studies Dermatokinetic study Skin irritation study	Showed spherical vesicles Sustained drug release Enhanced dermal flux Enhanced penetration of drug-loaded NLC gel Safer topical administration of herbal medications	[142]

**Table 7:** Herbal nano formulations for skin diseases.

Plants	Approaches	Techniques	Skin Disease	Types of Study	Outcomes	Ref.
*Zataria multiflora* (carvacrol, thymol)	Nanostructured lipid carriers	Ultrasonic probe	Cutaneous dermatophytosis	Particle size and zeta potential Cytotoxicity of NLCS In vitro antifungal susceptibility testing Clinical study of formulated topical gel	More effective cure for dermatophytosis Lower bioaccumulation/toxicity with broad-spectrum antifungal properties Anti-inflammation, size of lesion, itching, and scaling Improved antifungal activity	[146]
Aloe vera	Nanoflowers	-----	Wound healing	Scanning electron microscopy X-ray spectroscopy Fourier transform infrared spectroscopy X-ray diffraction In vitro wound healing	Highest peroxidase-mimicking activity DPPH assay determined the antioxidant activity Showed antimicrobial activity Wound healing Enhanced biological properties	[147]
*Sideroxylon mascatense* (quercetine, berberine, and myricetin)	Synthetic gel	---	Wound healing	Mean droplet size PDI Zeta potential pH Viscosity Spreadability	Enhanced efficacy Stability Bioavailability Higher antibacterial activity Low to moderate antifungal activity Reduced blood glucose level Increased the wound contraction rate Enhanced therapeutic potential	[148]
*Phyllanthus emblica* L. (sinapic and ferulic acid)	Topical gel	Rotary Evaporator	Anti-aging	In vitro assays Cellular assays Statistical analysis	Antioxidant, anti-tyrosinase, and anti-melanogenesis Anti-skin aging activities Improving skin hydration and elasticity, lightening the tone of the skin, and reducing wrinkles	[149]
*Olea europaea* and *Spirodela polyrhiza* (oleuropein, luteolin, and apigenin)	Topical oil	----	Atopic dermatitis	Atopic dermatitis symptoms Serum IgE levels Level of cytokine Gene expression in the dorsal skin Splenocytes Performed histological and immune cell subtype analyses	Controlling skin barrier function and immunological balance A potent treatment for atopic dermatitis Reduced epidermal thickness	[150]
Berberine	Gel-core oleosomes	Modified ethanol injection technique	Vitiligo	Ex vivo studies In vivo pharmacodynamic studies	Showed antioxidant and anti-inflammatory activity Sodium hyaluronate and sodium oleate both showed excellent skin penetration Sustained release of 45% at 24 h Higher stability Minimal systemic side effects	[151]
*Azadirachta indica*	Nanocapsule	Sonochemical method	Bacterial diseases	In vitro antibacterial activity Bacterial strain In vitro antibacterial activity	Immunological, anti-inflammatory, and anti-ulcer characteristics Antioxidant, antifungal, antibacterial, and antiviral properties Improved bioactivity and chemical activity High solubility Effective coating of neem extract Maximizing the aquaculture industry	[152]

**Table 8:** Patents on herbal formulations for skin diseases.

Title	Patent No.	Publication Date	Current Assignee	Ref.
Herbal nanoformulations for treating psoriasis and other skin conditions	WO2017172648A1	5 October 2017	Sirbal Ltd.	[153]
Topical nano liposome formulation, including extracted purified herbal mixture whitening cosmetics using this formulation	KR20050117958A	15 December 2005	SK Chemicals Co. Ltd.	[154]
Cosmetic composition for preventing skin aging comprising nano liposome of *Torilis japonica* fruit	KR100733334B1	29 June 2007	KT&G Co., Ltd., Korea Ginseng Corporation Co., Ltd.	[155]
Nano-sponge loaded topical gel of Curcumin and babchi oil for enhanced treatment of psoriasis.	DE202023101592U1	2 May 2023	Individual	[156]
Anti-acne nano preparation, gel composition, and preparation method thereof	CN115634165A	24 January 2023	Jiangsu Jicui New Pharmaceutical Preparation Technology Research Institute Co. Ltd.	[157]
Compound traditional Chinese medicine nano gel for treating dermatophytosis and tinea pedis and preparation method thereof	CN115300576A	8 November 2022	Changsha Medical University	[158]
Natural gel preparation of traditional Chinese medicine compound extract coated by nanoparticles, preparation method, and application thereof	CN115337282A	15 November 2022	Individual	[159]
Rigida pine bark extract with improved stability encapsulated nanoparticles and manufacturing method, a cosmetic composition comprising the same	KR20220152865A	17 November 2022	Kim Yu-mi and Jang Ki-hyeon	[160]
In-situ Gel Extraction, Formulation and Evaluation for Treating Fungal Skin Infection	AU2021107001A4	16 December 2021	Bhati Priyanka Ms. Khatoon Rizwana Mrs. Kumar Amrish Dr. Rahate Kalpana Dr. Sharma Akhil Dr. Sharma Shaweta Dr. Singh Veena Dr. Singh Vijay Dr. Sudha Anjali Ms	[161]
Novel *Punica granatum* Extracts-Zinc oxide Nanoparticles and its use	KR20220117942A	25 September 2023	Yeungnam University Industry-Academic Cooperation Foundation	[162]
Natural polysaccharide Nano-hydrogel mask based on hollow nanoparticles preparation method thereof	CN114010555A	8 February 2022	Luoyang Normal University	[163]
Berberis extract nanoformulation and process of preparation thereof	WO2022168124A1	11 August 2022	Panjab University, Chandigarh	[164]
Nanoparticle system for treating skin diseases and preparation method and preparation thereof	CN113081948A	9 July 2021	Anhui University of Traditional Chinese Medicine AHUTCM	[165]
Cosmetic preparation for the care and treatment of facial skin	DE202021106363U1	15 December 2021	Jassen GmbH	[166]

## Data Availability

Data can be made available on request to the corresponding authors.
